# Immune Gene Expression and Locomotor Activity in Response to *Vairimorpha ceranae* Infection Across Five Honey Bee Subspecies

**DOI:** 10.3390/insects16060593

**Published:** 2025-06-05

**Authors:** Cansu Özge Tozkar, Jay D. Evans

**Affiliations:** 1 Department of Agricultural Biotechnology, Faculty of Agriculture, Van Yüzüncü Yıl University, 65090 Van, Türkiye; 2 USDA-ARS Bee Research Laboratory, 10300 Baltimore Ave, BARC-East Bldg. 306 Rm 313, Beltsville, MD 20705, USA; jay.evans@usda.gov

**Keywords:** *Vairimorpha ceranae*, antimicrobial peptides (AMPs), locomotor activity, *Apis mellifera* subspecies, vitellogenin, *eater* gene

## Abstract

This study evaluated immune gene expression and locomotor behavior across five *Apis mellifera* subspecies (Carniolan, Caucasian, Syrian, Muğla ecotype, and Yığılca ecotype) following controlled *Vairimorpha ceranae* infection. Six days post-infection, Caucasian, Carniolan, and Yığılca bees exhibited a significant upregulation of antimicrobial peptide (AMP) transcripts—hymenoptaecin, abaecin, defensin, and apidaecin—indicating a robust humoral response. Conversely, Syrian and Muğla bees showed weaker AMP expression and higher *V. ceranae* mRNA levels, indicating lower immunity and higher susceptibility. Positive correlations among AMP transcripts, especially in Caucasian, Carniolan, and Yığılca bees, suggested a coordinated response. *Eater* gene expression, critical for cellular immunity, decreased in infected Caucasian and Yığılca bees, coinciding with AMP upregulation. Vitellogenin expression, linked to immunity and longevity, increased in Carniolan and Syrian bees, correlating with higher early locomotor activity. Locomotor analysis revealed subspecies-specific behavioral responses. Syrian bees maintained the highest activity despite elevated *V. ceranae* mRNA and minimal AMP expression, suggesting unique resilience possibly mediated by vitellogenin. Muğla bees, despite high pathogen loads, exhibited decreased activity. Caucasian bees showed strong immune responses but reduced activity post-infection, reflecting potential physiological trade-offs. Overall, these findings underscore the role of genetic variability in shaping honey bee immune and behavioral responses to *Vairimorpha* and support subspecies-targeted breeding and disease management strategies to enhance resilience.

## Simple Summary

Honey bees play a critical role in food production and environmental health, yet they are increasingly threatened by parasites like *Vairimorpha ceranae*. This microscopic organism compromises bee health by interfering with immune defenses and altering behavior. In our study, we explored how five honey bee subspecies responded to infection, focusing on immune gene activity and movement patterns. Subspecies such as Caucasian, Carniolan, and Yığılca showed strong immune gene activation, suggesting a more effective defense. In contrast, Syrian and Muğla bees had weaker immune responses and higher parasite levels, indicating greater vulnerability. Notably, some bees remained active even under infection, potentially compensating through alternative resilience mechanisms. Others showed reduced activity, likely reflecting physiological stress or energy limitations. These contrasting patterns highlight the diversity of biological responses among subspecies. Overall, our findings show that genetics matters—different bee types react differently to infection. Understanding this can guide local breeding programs and improve disease management to protect bee populations. This work is important for keeping healthy bees that can continue to support agriculture and nature.

## 1. Introduction

### 1.1. Vairimorpha (Formerly Nosema) Disease

Nosemosis is a critical disease affecting *Apis mellifera* worldwide [1]. It is driven by two species of microsporidia: *Vairimorpha apis* (formerly *Nosema apis*) and *V. ceranae* (*formerly Nosema ceranae*). Microsporidia comprise over 160 genera and 1300 species [2]. While *V. apis* was historically the predominant pathogen, *V. ceranae* has now emerged as the dominant species in many regions [3,4,5,6,7,8,9,10,11,12]. 

*Vairimorpha* spreads throughout the colony via the fecal–oral route, involving contaminated food, water, feces, or combs. The metabolically inactive spores germinate in the bee’s midgut leading to the production of millions of spores within weeks [1]. Infections caused by *Vairimorpha* result in a range of negative effects, including dysentery, shortened lifespans, queen replacement, reduced colony size, high winter mortality, poor pollen collection, decreased honey production, and diminished crop yields reliant on bee pollination [13,14]. *V. ceranae* specifically disrupts the physiological functions and behavioral patterns of honey bees, resulting in low carbohydrate levels, increased appetite, and altered food-sharing behavior in infected foragers, which negatively impacts food intake and survival [15,16,17]. The research indicates that *V. ceranae* exhibits greater virulence than *V. apis* in cage trials as well as in natural settings [3,9,18]. This pathogen has also been linked to Colony Collapse Disorder (CCD) in the U.S. and Honey Bee Colony Depopulation Syndrome (CDS) in Europe, leading to significant economic and ecological impacts worldwide [19,20]. 

### 1.2. Honey Bee Subpopulations

Turkey harbors five *A. mellifera* subspecies: *A. mellifera carnica*, *A. mellifera anatoliaca*, *A. mellifera caucasica*, *A. mellifera syriaca*, and *A. mellifera meda*. Carniolan honey bees (*A. mellifera carnica*) are found in Thrace, while *A. mellifera syriaca* is located in Hatay, and *A. mellifera caucasica* resides in northeastern Anatolia, particularly in Ardahan and Artvin near the Georgian border [21]. *A. mellifera anatoliaca* is distributed across various regions of Anatolia, with regionally adapted varieties in areas like Muğla, Giresun, and Yığılca [22]. 

### 1.3. Honey Bee Locomotion

Ref. [23] reported that circadian cycles in honey bees regulate physiological and behavioral mechanisms, influencing colony structure. Nurse bees care for the brood and perform hive tasks without rhythmicity for the first two-to-three weeks of adulthood. As adult bees transition from in-hive tasks to foraging, their circadian rhythms become more pronounced and they are exposed to strong zeitgebers due to increased time outside the dark, temperature-controlled hive. Ref. [24] found low levels of mRNA for the “clock protein” Per in immature bees, whereas rhythmic foragers had higher levels of this mRNA. Foragers that reverted to nursing exhibited arrhythmic activity but still maintained high *per* mRNA levels [25]. Bee foraging behavior aligns with daily floral cycles to maximize efficiency and conserve energy, with rest periods inside the hive away from intense activity. Social factors, including the division of labor, influence the temporal activity patterns of juvenile bees, affecting their physiology, behavior, and circadian rhythms [26,27]. 

Brain mRNA levels in foragers fluctuate throughout the day, while they remain constant in nurse bees, indicating continuous activity in brood care. Various methods have been used to study behavioral cycles in isolated solitary bees [28]. Although circadian rhythms are crucial for social insects, the research on these rhythms in honey bees and bumblebees has significantly expanded since the early 2000s [24,25,29,30,31]. Over 600 studies [32,33,34,35] have used the DAM system, with [36] confirming its effectiveness in determining honey bee circadian cycles.

### 1.4. Honey Bee Immunity

Honey bees use behavioral, physical, physiological, and immunological strategies to combat pathogens and parasites [37]. Initial barriers include the exoskeleton cuticle, peritrophic membranes, along with alterations in pH levels and digestive chemical compounds [38]. The second line of defense involves cellular and humoral immunity [39,40]. The immune response at the cellular level involves mechanisms such as phagocytosis, encapsulation, and melanization, which are mediated by enzymes, such as phenol oxidase (PO) and glucose dehydrogenase (GLD) [41,42,43]. Another innate immune response involves the production of melanin and quinones, triggered by prophenoloxidase (proPO) activation [44]. *Eater* is essential for bacterial detection and the phagocytic process in *Drosophila* [45,46]. The humoral immune response includes the production of antimicrobial defense peptides (AMPs) as a reaction to bacterial, fungal, and parasitic infections [47]. Honey bees produce AMPs such as abaecin, apidaecin, defensin, and hymenoptaecin [48,49,50]. Vitellogenin (Vg) in honey bees regulates immunity, lifespan, and reproductive function. Its expression is also linked to labor division, foraging specialization, and resistance to oxidative stress [51,52,53]. *A. mellifera* genome has four immunological pathways that respond to *Vairimorpha* infection: the Toll, IMD, c-Jun N-terminal kinase (JNK), and Janus kinase/signal transducer and activator of transcription (JAK/STAT) pathways. The Toll signaling pathway plays a key role in protection from fungi and Gram-positive bacteria, whereas the IMD pathway plays a key role in combating Gram-negative bacterial infections [54]. 

This research sought to examine how *V. ceranae* infection influences the immune response and locomotor activity in five *A. mellifera* subspecies. Immune gene expression levels were assessed six days post-infection to evaluate antimicrobial peptide (AMP), *eater*, and vitellogenin (Vg) responses. Locomotor activity was monitored over a 12-day period using the Drosophila Activity Monitoring (DAM) system, with daily activity data analyzed for the first six days to allow a direct comparison with immune responses. Additionally, cumulative 12-day activity data were included to provide a broader perspective on infection-related behavioral changes. Given the genetic variability among *A. mellifera* subspecies, this study provides insights into the interplay between immune activation, behavioral changes, and subspecies-specific resilience to *V. ceranae* infection, contributing to a better understanding of host–pathogen dynamics and potential mechanisms of disease resistance in honey bees.

## 2. Materials and Methods

### 2.1. Collection of Worker Bees for V. ceranae Infection Assays

**Figure 1:**
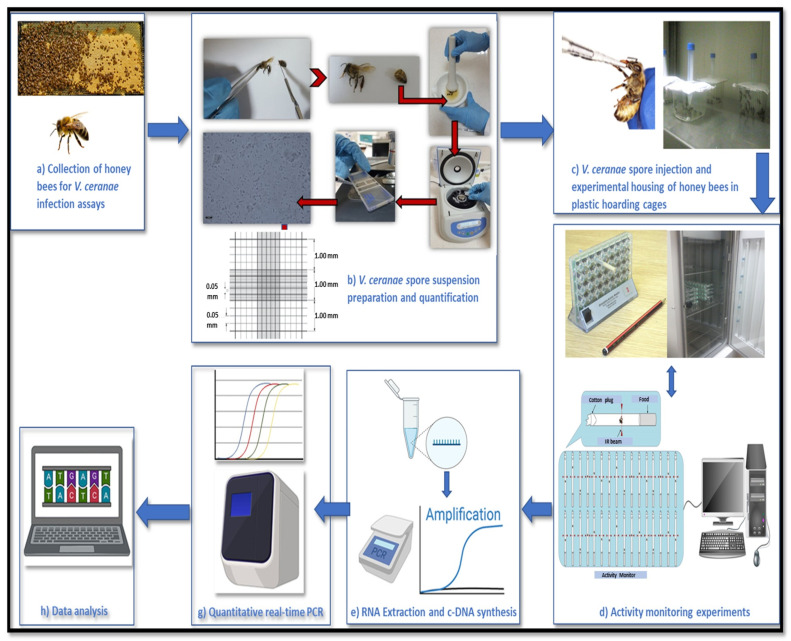
Experimental workflow for *Vairimorpha* (*Nosema*) *ceranae* infection, locomotor activity monitoring, and gene expression analysis in honey bees. The workflow includes (**a**) honey bee collection, (**b**) *V. ceranae* spore preparation and quantification (obtained from [55], (**c**) infection and experimental housing, (**d**) locomotor activity monitoring, (**e**) RNA extraction and cDNA synthesis, (**g**) quantitative real-time PCR (qPCR), and (**h**) data analysis.

Ten thriving colonies representing *A. m. carnica*, *A. m. syriaca*, *A. m. caucasica*, along with the Muğla and Yığılca ecotypes of *A. m. anatoliaca*, were sampled from a region accessible on the METU campus in Ankara. Brood frames were taken from each colony, and two colonies per subspecies were used as biological replicates (Figure 1a). The developing brood was incubated at 34 ± 1 °C under 55% humidity to allow the emergence of adult bees, which were examined with light microscopy to confirm the absence of visible *Vairimorpha* disease symptoms.

### 2.2. Vairimorpha ceranae Spore Suspension Preparation and Quantification

Midguts from 20 bees severely infected with *Vairimorpha* were dissected with forceps. These midguts were processed in 1 mL of distilled water through homogenization and the suspension was spun at 6000–10,000× *g* rpm for 5–10 min. The pellet was dissolved in 1 mL of water, and spore concentrations were calculated using a Leica DM300 light microscope (Leica Microsystems, Wetzlar, Germany) and Neubauer cell-counting grid (Marienfeld Superior, Lauda-Königshofen, Germany) (Figure 1b). A micropipette was used to deliver 5 µL of sucrose solution with a concentration of 10,000 *V. ceranae* spores to one-day-old bees. Bees that consumed the entire droplet were considered infected, while those that did not were eliminated. The control group received 5 µL of sucrose mixture free of spores. Worker bees (*n* = 20 bees per colony) were housed within plastic confinement cages, each containing a sterile feeder stocked with 50% *w*/*v* sucrose solution, and kept at 34 ± 1 °C under 55% humidity (Figure 1c).

### 2.3. Activity Monitoring Experiments

The locomotor activity of five *A. mellifera* subspecies was monitored for 12 days post-infection using the Drosophila Activity Monitoring (DAM) system. Each activity monitor had 32 independent channels, with three infrared beams and sensors ensuring accurate data collection. Bees were placed individually in 15 mL falcon tubes and monitored for movement using infrared sensors. Movement data were recorded continuously for 24 h, resulting in 1440 measurements per bee. Data were collected using the DAM System 3.03 (TriKinetics Inc., Waltham, MA, USA). Readings of temperature, humidity, and light intensity were taken every minute using an environmental monitor to ensure consistency. Monitoring took place inside an incubator set to 34 ± 1 °C, maintained at 55% relative humidity and constant darkness to mimic natural hive conditions. In total, 120 honey bees (60 infected, 60 controls) were monitored, with 12 bees per condition for each subspecies. Bees were nourished with a solid sucrose-based feed, covered with cheesecloth to prevent attachment, and all tubes had four holes for air circulation (Figure 1d).

It should be noted that individual isolation for activity monitoring, while necessary for standardized measurements, removes social context that would naturally influence bee behavior in a colony setting. This methodological approach was deliberately chosen to enable direct comparisons across subspecies and treatment groups by eliminating confounding social interactions, though we acknowledge this represents a simplification of the complex social environment that regulates honey bee behavior under natural conditions.

### 2.4. RNA Extraction, Quantification, cDNA-Synthesis, and qPCR

For immune gene expression studies, RNA from 12 control and 12 infected bees of each of the five subspecies was isolated using the TRIzol^®^ (Invitrogen, Carlsbad, CA, USA) technique [56] and quantified with a Nanodrop ND-1000 (Thermo Fisher Scientific, Waltham, MA, USA). Whole honey bee homogenates were used for RNA extraction. This approach was chosen to obtain a comprehensive profile of systemic immune responses while maintaining consistency with our experimental workflow, particularly given that the same individuals were subjected to behavioral assessments before tissue collection.

According to [57], RNA extracts were used to synthesize cDNA with random hexamer primers and the Superscript II^®^ (Invitrogen™) enzyme for reverse transcription (Figure 1e). Previously established primers for *V. ceranae*, apidaecin, abaecin, defensin, hymenoptaecin, *eater*, and *vitellogenin* genes were utilized [47,58]. Honey bee ribosomal protein S5 (RPS5) and β-actin served as reference genes to normalize cDNA content and screen samples for degradation or experimental loss [47]. 

Quantitative real-time PCR (qPCR) was performed on a Bio-Rad CFX-96 thermocycler under a thermal profile of 95 °C for 30 s, followed by 95 °C for 5 s and 60 °C for 30 s, for a total of 50 cycles, with fluorescence recorded at each 60 °C step to quantify pathogen loads. Following amplification, products were denatured for 10 s at 95 °C, then reannealed, and dissociation profiles were monitored between 69 °C and 95 °C at 0.5 °C increments [47] (Figure 1g). Each 96-well plate included both positive and negative control reactions. Pathogen loads (ΔΔCT = CTcontrol − CTtarget) were calculated as the difference between the CT of actin and the CT of each target (ΔCT), adjusted relative to the smallest ΔCT value across all samples [47,59].

The timepoints for immune gene analysis and activity monitoring were selected based on the literature and preliminary experiments. The 6-day timepoint for immune gene expression was chosen as it represents a critical window for detecting robust *V. ceranae* immune responses in honey bees. Studies by [60,61,62] documented significant AMP expression changes at this timepoint. This period precedes peak spore production (typically 7–10 days post-infection), allowing the capture of active immune response rather than potential immunosuppression during later infection stages.

The 12-day locomotor monitoring period was selected to observe behavioral changes throughout both early- and late-infection phases. This extended monitoring was essential for detecting delayed behavioral effects not apparent during initial infection. We analyzed day-6 activity patterns alongside immune gene expression for direct comparison while collecting complete 12-day data to understand comprehensive temporal infection dynamics. This dual-timepoint approach provided insights into both immediate behavioral correlates of immune activation and longer-term effects of infection progression on locomotor patterns.

### 2.5. Statistical Analysis

Descriptive statistics for immune gene expression and activity monitoring were computed with JMP™ (v.9, SAS Institute, Cary, NC, USA). Graphs were used to illustrate relationships between variables. The Analysis of Variance (ANOVA) report served as a tool for hypothesis testing. A single-factor Analysis of Variance (ANOVA) was performed to evaluate differences among group means, with variability partitioned into within-group and between-group components. Multiple comparisons were performed based on combined variance estimates, and pairwise comparisons were conducted using Student’s *t*-tests. Pearson correlation coefficients were computed to assess linear relationships between response variables, with significance determined using the pairwise deletion method (Figure 1h).

## 3. Results

**Figure 2:**
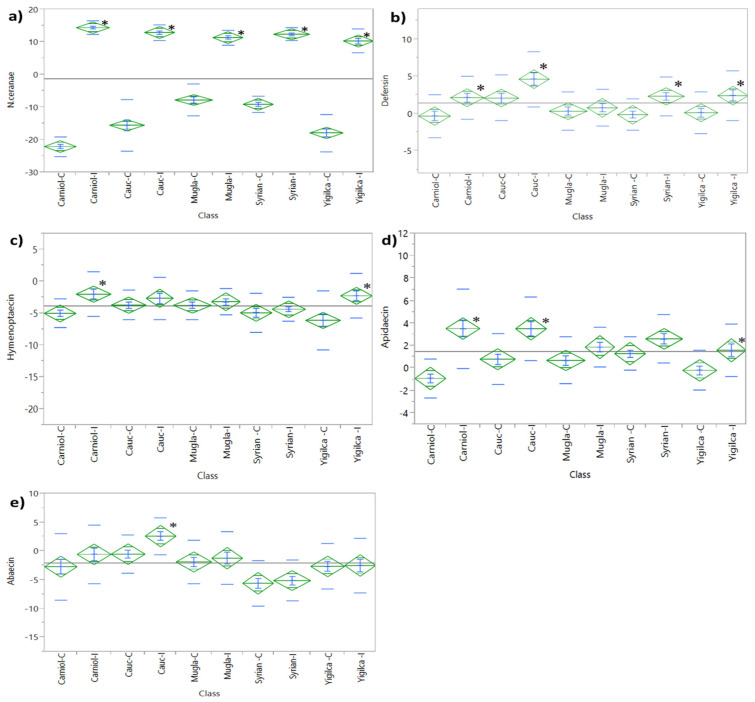
One-way Analysis of Variance (ANOVA) of (**a**) *V. ceranae* mRNA expression, (**b**) defensin, (**c**) hymenoptaecin, (**d**) apidaecin, and (**e**) abaecin across different *A. mellifera* subspecies (Carniolan, Caucasian, Muğla, Syrian, and Yığılca). “C” represents control groups and “I” represents infected groups. Asterisks (*) indicate significant differences (*p* < 0.0001) between infected and control groups.

*Vairimorpha ceranae* mRNA expression levels were significantly higher in infected groups compared to the controls across all analyzed subspecies (Figure 2a; *t*-test, *p* < 0.05 for all comparisons). When considering the mean *V. ceranae* mRNA expression across both the infected and control groups, the Syrian subspecies exhibited the highest expression levels, whereas the Yığılca subspecies had the lowest. This difference between these two subspecies was statistically significant (*t*-test, *p* = 0.0239). The Muğla, Caucasian, and Carniolan subspecies followed the Syrian race with ascending degrees of *V. ceranae* expression, however the differences among them were not statistically significant. A two-way ANOVA (SS = 2012.09, *F* ratio = 28.96, *p* < 0.0001) confirmed significant effects of both race and treatment on *V. ceranae* mRNA expression, with a significant interaction between these two factors (*p* < 0.0001).

### 3.1. Immune Gene Expressions

#### 3.1.1. Defensin Expression

*V. ceranae* challenge resulted in an upregulated expression of defensin (ANOVA, *p* < 0.0001; Figure 2b). When considering the mean defensin mRNA expression across both the infected and control groups, the Caucasian subspecies exhibited the highest overall expression levels (*p* = 0.0004). An increasing trend in defensin transcription was observed in the Caucasian, Carniolan, Syrian, and Yığılca subspecies six days after *V. ceranae* inoculation (*t*-test, *p*-values = 0.0042, 0.0053, 0.0048, and 0.0102, respectively). However, within each subspecies, infected bees consistently exhibited higher defensin expression compared to their respective controls, except for Muğla bees, where no significant differences were detected between control and infected groups.

Two-way ANOVA revealed significant associations between defensin transcript levels and both race (SS = 211.2, *F* ratio = 6.5, *p* < 0.0001) and treatment (SS = 223.9, *F* ratio = 27.4, *p* < 0.0001). A significant correlation between defensin and *V. ceranae* transcripts was observed in Carniolan, Syrian, and Yığılca subspecies (*r* = 0.3939, *p* = 0.0068; 0.4451, *p* = 0.0048; and 0.3895, *p* = 0.0102, respectively). Additionally, defensin levels were significantly correlated with apidaecin in Carniolan (*r* = 0.7411, *p* < 0.0001), Caucasian (*r* = 0.7299, *p* < 0.0001), Syrian (*r* = 0.6798, *p* < 0.0001), Muğla (*r* = 0.3970, *p* = 0.0052), and Yığılca (*r* = 0.7411, *p* < 0.0001) subspecies.

#### 3.1.2. Hymenoptaecin Expression

Exposure to *V. ceranae* led to a notable upregulation of hymenoptaecin expression in treated bees compared to controls within the subspecies, as evaluated through ANOVA (*p* < 0.0001; Figure 2c). Specifically, a notable increase in hymenoptaecin expression was observed in *V. ceranae*-infected Yığılca bees (*t*-test, *p* < 0.0001) and Carniolan bees (*t*-test, *p* = 0.0013).

A two-way ANOVA revealed a significant interaction between subspecies and treatment effects on hymenoptaecin expression (SS = 96.1, *F* = 2.7, *p* = 0.0338). Expression levels of hymenoptaecin were more significantly influenced by *V. ceranae* exposure than by race (SS = 168.3, *F* = 18.6, *p* < 0.0001). A strong correlation was identified between hymenoptaecin and abaecin in the Carniolan (*r* = 0.3222, *p* = 0.0290), Caucasian (*r* = 0.3786, *p* = 0.0095), Syrian (*r* = 0.3210, *p* = 0.0296), and Yığılca (*r* = 0.3848, *p* = 0.0091) subspecies. Similarly, hymenoptaecin and defensin were strongly correlated in Carniolan (*r* = 0.8429, *p* < 0.0001), Caucasian (*r* = 0.3562, *p* = 0.0151), Syrian (*r* = 0.8310, *p* < 0.0001), Yığılca (*r* = 0.7411, *p* < 0.0001), and Muğla (*r* = 0.5234, *p* = 0.0001) subspecies.

#### 3.1.3. Apidaecin Expression

A pronounced upregulation in apidaecin expression was detected in infected Carniolan (*p* < 0.0001), Caucasian (*p* = 0.0002), and Syrian (*p* = 0.0299) populations, as well as in Yığılca populations (*p* = 0.0104). In contrast, the increase in the Muğla population was non-significant (Figure 2d). When considering the mean apidaecin expression, the Caucasian subspecies exhibited the highest overall expression levels, whereas the Yığılca subspecies showed the lowest.

Two-way ANOVA revealed significant effects of both race (SS = 57.0, *F* = 2.7, *p* = 0.0302) and treatment (SS = 279.0, *F* = 53.4, *p* < 0.0001), along with a notable interaction between race and treatment (SS = 76.5, *F* = 3.6, *p* = 0.0066). Apidaecin and *V. ceranae* transcripts showed significant correlations in Carniolan (*r* = 0.5674, *p* < 0.0001), Caucasian (*r* = 0.3732, *p* = 0.0106), Syrian (*r* = 0.3790, *p* = 0.0094), and Yığılca (*r* = 0.3158, *p* = 0.0346) populations. Additionally, strong associations between apidaecin and hymenoptaecin transcripts were observed in Carniolan (*r* = 0.7897, *p* < 0.0001), Caucasian (*r* = 0.3412, *p* = 0.0203), Syrian (*r* = 0.7118, *p* < 0.0001), Yığılca (*r* = 0.6507, *p* < 0.0001), and Muğla (*r* = 0.5234, *p* = 0.0001) populations.

#### 3.1.4. Abaecin Expression

Abaecin expression increased following *V. ceranae* infection in all subspecies (Figure 2e). Although this rise was noticeable in Carniolan, Caucasian, and Muğla populations, ANOVA revealed a statistically significant upregulation only in the Caucasian subspecies (*t*-test, *p* = 0.0185).

Two-way Analysis of Variance revealed that race (SS = 909.6, *F* = 12.5, *p* < 0.0001) and treatment (SS = 92.1, *F* = 5.0, *p* = 0.0255) independently influenced abaecin expression. Significant positive correlations were identified between abaecin and defensin across Carniolan (*r* = 0.3658, *p* = 0.0124), Caucasian (*r* = 0.4984, *p* = 0.0004), Muğla (*r* = 0.2923, *p* = 0.0438), and Yığılca (*r* = 0.5014, *p* = 0.0005) colonies. Similarly, abaecin and apidaecin showed notable correlations in Carniolan (*r* = 0.4042, *p* = 0.0053), Caucasian (*r* = 0.6396, *p* < 0.0001), Yığılca (*r* = 0.4368, *p* = 0.0027), and Muğla (*r* = 0.3255, *p* = 0.0240) populations.

#### 3.1.5. Eater Expression

**Figure 3:**
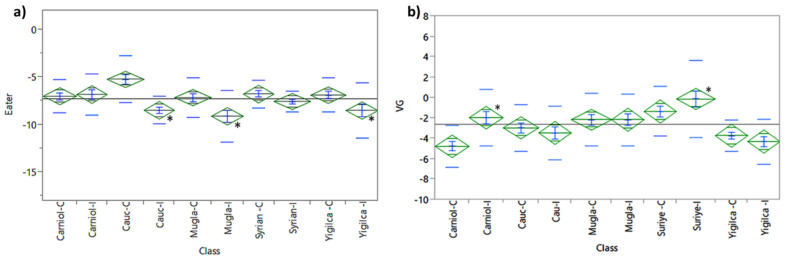
One-way Analysis of Variance (ANOVA) of (**a**) *eater* and (**b**) vitellogenin (VG) expression levels across different *A. mellifera* subspecies following *V. ceranae* infection. “C” represents control groups and “I” represents infected groups. Asterisks (*) indicate statistically significant differences between control and infected groups (*p* < 0.0001).

In *V. ceranae*-injected bees, *eater* transcript levels were significantly reduced compared to the controls in Caucasian (*p* < 0.0001), Muğla (*p* = 0.0021), and Yığılca (*p* = 0.0117) populations, while no significant difference was observed in the Syrian population. For Carniolan bees, *V. ceranae* infection had no significant effect on *eater* gene expression (Figure 3a).

Two-way ANOVA revealed significant effects of race (SS = 50.1, *F* = 2.9, *p* = 0.0212), treatment (SS = 114.7, *F* = 27.0, *p* < 0.0001), and their interaction (SS = 67.6, *F* = 3.9, *p* = 0.0040). Strong correlations were identified between *eater* and *vitellogenin* transcript levels in Caucasian (*r* = 0.3861, *p* = 0.0080), Syrian (*r* = 0.4856, *p* = 0.0006), and Yığılca (*r* = 0.3093, *p* = 0.0387) populations. Moreover, *eater* transcript levels showed strong correlations with *apidaecin* (*r* = 0.3904, *p* = 0.0080), *defensin* (*r* = 0.3525, *p* = 0.0176), and *hymenoptaecin* (*r* = 0.3318, *p* = 0.0260) in Yığılca bees, while similar associations were observed for *apidaecin* in Caucasian bees (*r* = 0.4970, *p* = 0.0004).

#### 3.1.6. Vitellogenin Expression

Overall, vitellogenin expression was highest in Syrian bees, followed by Muğla, Caucasian, and Carniolan subspecies, while Yığılca bees exhibited the lowest levels (ANOVA, *p* < 0.0001). However, when control (C) and infected (I) groups were examined separately, significant within-subspecies differences were observed. In particular, Syrian and Carniolan bees showed a notable increase in vitellogenin expression following *V. ceranae* infection, whereas Yığılca bees exhibited consistently low levels regardless of infection status (Figure 3b).

Two-way ANOVA revealed significant effects of race (SS = 279.8, *F* = 10.5, *p* < 0.0001) and treatment (SS = 86.1, *F* = 3.2, *p* = 0.0132) on vitellogenin expression levels, with a notable interaction between these two factors. Positive correlations were observed between vitellogenin transcripts and *V. ceranae* infection in Carniolan (*r* = 0.4296, *p* = 0.0029) and Syrian bees (*r* = 0.3408, *p* = 0.0205). However, no significant correlations were detected in other subspecies. These findings highlight the significant role of race in regulating vitellogenin expression levels, as evidenced by the strong association between race and vitellogenin expression in the two-way ANOVA (SS = 279.8, *F* = 10.5, *p* < 0.0001).

### 3.2. Honey Bee Locomotion

**Figure 4:**
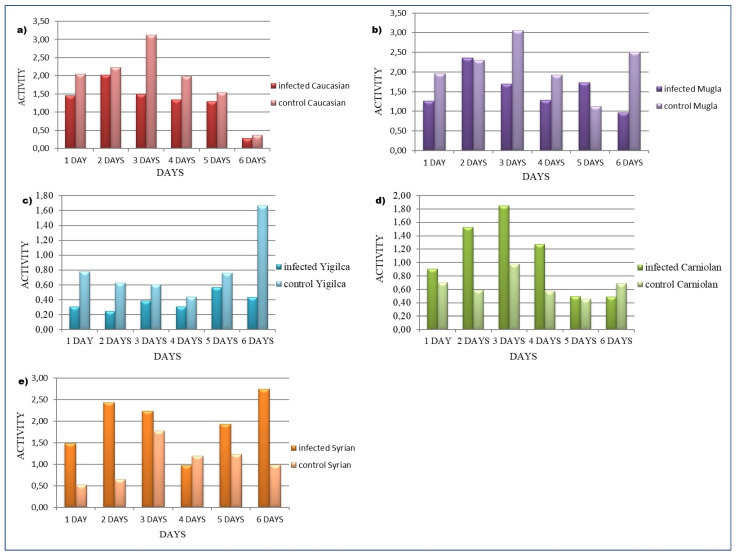
Locomotor activity patterns in five *A. mellifera* subspecies over a 6-day period following *V. ceranae* infection. The graphs display the activity levels of infected (darker bars) and control (lighter bars) bees from Caucasian, Muğla, Yığılca, Carniolan, and Syrian subspecies. Each bar represents the mean activity level at different timepoints. Activity levels shown for (**a**) Caucasian, (**b**) Muğla, (**c**) Yığılca, (**d**) Carniolan, and (**e**) Syrian subspecies. Infected groups are shown in darker bars and control groups in lighter bars. Each bar represents the mean activity level at different timepoints (1–6 days post-infection).

In both Muğla and Caucasian subspecies, *V. ceranae*-infected bees generally showed lower activity levels than the controls. In Caucasian control bees, activity peaked on day 3 before exhibiting a declining trend. In Muğla control bees, activity also peaked on day 3 but displayed a slight secondary increase on day 6. Infected bees in both subspecies generally maintained lower activity levels, with infected Caucasian bees showing a minor increase on day 2 and infected Muğla bees exhibiting moderate increases on days 2 and 5 (Figure 4a,b).

In contrast, the Yığılca ecotype exhibited a different trend. Control bees showed a notable increase in activity on day 6, diverging from the relatively stable pattern observed in the previous days. Infected Yığılca bees maintained lower activity levels than controls, but small fluctuations were observed throughout the period, though no distinct peak was recorded (Figure 4c). Infected Carniolan bees initially exhibited higher activity than controls, but this difference diminished by day 5 and reversed on day 6 (Figure 4d). Similarly, infected Syrian bees showed higher activity levels than controls until day 6, after which activity declined significantly (Figure 4e). Data beyond day 6 are not included in the analysis shown.

**Figure 5:**
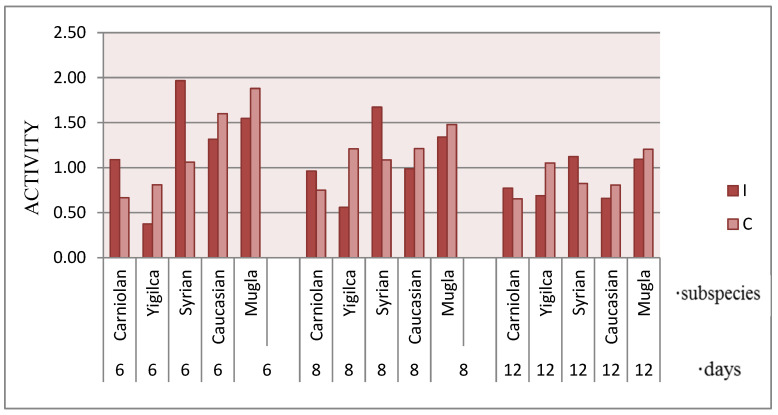
Cumulative locomotor activity patterns of honey bee subspecies on days 6, 8, and 12 following *V. ceranae* exposure. Bars represent cumulative activity levels for infected (I) and control (C) groups across different subspecies.

Cumulative activity data reveal significant differences across subspecies when comparing untreated and *V. ceranae*-exposed bees over a 12-day period (ANOVA: *F* = 2.8015, *p* = 0.0369) (Figure 5). On day 6, infected Syrian bees exhibited the greatest activity, with Muğla, Caucasian, Carniolan, and Yığılca bees displaying progressively lower levels. By day 8, activity levels declined across all infected subspecies except for infected Yığılca (F = 2.7964, *p* = 0.0354).

With the exception of the Yığılca ecotype, all subspecies exhibited a downward trend in activity levels during the monitoring period. While cumulative data suggest an increase in Yığılca activity, daily measurements indicate minimal variability in activity rates. Control bees of Muğla, Caucasian, and Yığılca subspecies exhibited higher activity rates than infected bees. Conversely, cumulative activity data suggest higher mean activity in infected Carniolan and Syrian bees. However, daily monitoring revealed that this pattern reversed after day 6, with infected groups exhibiting lower activity than controls.

Race significantly influenced cumulative activity data during the first 8 days of infection (day 1: *p* = 0.0164; day 2: *p* = 0.0082; day 3: *p* = 0.0088; day 4: *p* = 0.0037; day 5: *p* = 0.0030; day 6: *p* = 0.0061; day 7: *p* = 0.0393).

## 4. Discussion

### 4.1. Immune Gene Responses

In this study, immune responses in honey bees were rapidly triggered to counter *V. ceranae* in all five studied subspecies. Caucasian, Carniolan, and Yığılca bees showed significant increases in hymenoptaecin, abaecin, defensin, and apidaecin transcripts, indicating a strong antimicrobial peptide response. In contrast, Syrian and Muğla subspecies had significantly higher *V. ceranae* mRNA levels, but their antimicrobial peptide expression was weaker, particularly in Muğla bees, where no significant defensin or apidaecin upregulation was detected. These findings suggest that Syrian and Muğla bees may have lower humoral immunity and a higher susceptibility to *V. ceranae* infection, although alternative immune pathways, such as vitellogenin-related mechanisms, might play a role in Syrian bees. Genetic variability influences immune responses, with differences in AMP expression intensity linked to genetic history [63,64]. Additionally, worker honey bees quickly raised their immunological responses to *V. ceranae* in experimental infection trials, according to [65,66,67]. Ref. [62] found an over-expression of some AMPs at 6 days p.i. following both *V. apis* and *V. ceranae* infections, while the effects of 14 days post-infection were not particularly noticeable [68]. However, in contrast to these findings, other studies have reported signs of immunosuppression following *V. ceranae* infection. For instance, Ref. [69] detected immunosuppression at some timepoints post-infection (p.i.) and immune upregulation at others. Similarly, after *V. ceranae* exposure, a partial suppression of the humoral defense mechanism was observed by [60]. Interestingly, these authors found that *V. apis* induced the expression of abaecin, defensin, and hymenoptaecin by four days p.i. [60]. AMPs were downregulated at day 5 post-infection (p.i.) but not at days 10 or 15 p.i in honey bees (*A. mellifera*) infected with a relatively high dose of 86,000 *V. ceranae* spores per individual, based on the RNA-seq analysis of gene expressions [70], suggesting a conditional immune response.

For each subspecies examined in this research, abaecin, defensin, apidaecin, and hymenoptaecin transcript levels exhibited significant positive correlations, particularly in Caucasian, Carniolan, and Yığılca bees. The significant correlation observed between hymenoptaecin and abaecin expression across multiple subspecies suggests a co-regulated antimicrobial peptide response to *V. ceranae* infection. This pattern, particularly evident in Carniolan, Caucasian, Syrian, and Yığılca subspecies, implies that these AMPs may act synergistically in combating the infection. In a study examining the vulnerability of four different *Apis* species to *V. ceranae* infection, *abaecin* expression levels remained unchanged in all four species. On the other hand, infection with a *V. ceranae* strain originating from Canada led to a remarkable upregulation of apidaecin in *A. cerana* and *A. florea*, as well as an increase in hymenoptaecin mRNA expression in *A. cerana* [61]. Additionally, apidaecin expression was significantly upregulated in queen bees of different ages after *V. ceranae* exposure (six days post-infection) [61]. 

This research identified a notable decrease in *eater* transcript abundance six days following *V. ceranae* exposure. While [71] reported no significant change in *eater* expression in bees exposed to *V. ceranae*, another study found that *eater* expression was downregulated in the stomach tissue of queen honey bees at different age points (1, 6, and 12 days old) six days post-exposure [61]. Eater is a transmembrane receptor with an EGF-like repeat, originally identified in the hemocytes of *Drosophila*. Its homolog within the *A. mellifera* genome plays a key role in phagocytosis of a wide range of bacterial invaders [46,47]. 

In this study, the humoral immune defense was triggered prior to the activation of cellular immune defense. After six days of *V. ceranae* exposure, *eater* transcript levels decreased particularly in Caucasian and Yığılca bees, coinciding with the upregulation of antimicrobial peptides. Further studies are needed to clarify the functional link between *eater* suppression and AMP activation. In bacterial infections, AMP release into the hemolymph by the fat body is known to enhance host resistance before eater-mediated phagocytosis occurs [72], suggesting a potential parallel mechanism in *V. ceranae* infections, though additional research is required to confirm this relationship. The Toll pathway has been identified as the primary immune response pathway against *V. ceranae* infection [65]. *Eater* has been shown to play a role in phagocytosis rather than directly influencing the Toll or IMD pathways in *Drosophila* [46]. In *E. coli* and fungal infections, *eater* expression did not contribute to the activation of these pathways, suggesting its involvement in alternative immune mechanisms.

*V. ceranae* infection significantly increased vitellogenin (Vg) expression in Carniolan and Syrian subspecies, demonstrating a robust association with *V. ceranae* transcript levels. These subspecies exhibited stronger cellular defenses, as Carniolan bees showed only a minimal, non-significant increase in *eater* expression, while Syrian bees displayed only a slight reduction in *eater* transcript levels. In contrast, other subspecies exhibited a more pronounced decline in *eater* transcript levels. Vitellogenin (Vg), a yolk protein essential for reproduction, also plays key roles in shielding cells against oxidative stress, modulating immune responses, and extending longevity [53,73]. In contrast to *A. cerana*, where Vg expression declined 14 days post-infection [68]. *A. mellifera* exhibited increased Vg levels at both 7 and 14 days after *V. ceranae* challenge. Under field conditions, lower Vg expression has been associated with reduced oxidative stress resistance and shorter lifespans [52,74]. Ref. [71] reported no notable alterations in vitellogenin (Vg) transcript levels after *V. ceranae* inoculation. However, some studies have reported contrasting findings about how *V. ceranae* infection influences Vg expression. While [60,70] observed impaired Vg expression associated with reduced worker bee lifespan—detecting significant effects on days 7 and 5 following infection, respectively—other studies documented an increase in Vg levels under specific conditions. In queen bees, Vg transcript levels increased in 1- and 12-day-old individuals but decreased in 6-day-old ones [61]. Ref. [75] observed a 58% rise in Vg levels in infected queens, while [76] recorded Vg titer increases of 83% and 73% in 1-day-old bees at different *V. ceranae* doses, with a 68% increase at day 7 in the 10K treatment.

Additionally, increased vitellogenin levels in 9- and 12-day-old bees from a colony with a low infection level may indicate a normal physiological response rather than a direct consequence of *V. ceranae* exposure, as vitellogenin transcript levels remained unchanged following inoculation. Similar findings were reported for antimicrobial peptides (AMPs) in the same study, where the authors suggested that differentially expressed vitellogenin could be linked to *V. ceranae* infection levels [77]. Vg has also been recognized as a marker for colony health, with no variation in collapsed colonies but a significant increase in those that survived [78]. Variability in honey bee immune responses across studies could arise due to variations in the genomic makeup of the host, *Vairimorpha* variants, spore dosages, feeding methods, tissue sources, or physiological factors.

The differential immune responses observed among subspecies, particularly the weaker AMP expression coupled with higher pathogen loads in Syrian and Muğla bees, present an intriguing immunological paradox. Four non-mutually exclusive hypotheses may explain these subspecies-specific patterns. These subspecies may rely on alternative immune defense mechanisms beyond canonical AMP production. The elevated vitellogenin expression in Syrian bees supports this hypothesis, as vitellogenin functions as a potent antioxidant enhancing resilience against oxidative stress induced by infection [53,74]. The positive correlation between vitellogenin transcripts and *V. ceranae* infection in Syrian bees (*r* = 0.3408, *p* = 0.0205) suggests the adaptive regulation of this protein in response to infection. These differences may reflect evolutionary trade-offs between resistance and tolerance. Syrian bees maintained high locomotor activity despite substantial pathogen loads, suggesting evolved tolerance mechanisms that minimize fitness costs while permitting pathogen replication. This strategy could be adaptive if the energetic costs of AMP responses outweigh the benefits in certain ecological contexts [79]. The geographical origin of Syrian bees in warmer climates with different pathogen pressures may have favored tolerance over resistance strategies.

Resource allocation trade-offs may explain the inverse relationship between immune activation and locomotor activity. Immune responses are energetically costly [80] requiring resource partitioning between immunity, metabolism, and behavior. Infected Syrian bees maintained locomotor performance, suggesting prioritization of behavior over AMP production, while Caucasian bees showed strong immune upregulation but reduced activity, indicating energy re-allocation toward immune defense. Genetic constraints in immune pathway regulation may limit AMP induction in certain subspecies. Polymorphisms in regulatory regions of AMP genes or in signaling cascade components could create subspecies-specific immune activation thresholds [81]. Future research should test these hypotheses through: (1) comparative hemolymph proteomics to identify alternative immune effectors; (2) RNA interference of vitellogenin followed by survival assessment; (3) dose–response infection studies to distinguish resistance from tolerance; (4) metabolic measurements to quantify immune response energy costs; and (5) genomic analyses of immune pathway components, particularly transcription factors like Dorsal and Relish, and negative regulators like Cactus (47). These approaches would provide mechanistic insights into subspecies-specific immune responses, potentially identifying novel resistance or tolerance mechanisms for breeding programs.

### 4.2. Locomotion

The locomotor activity of five *A. mellifera* subspecies was monitored over a 12-day period after *V. ceranae* exposure using a Drosophila activity monitoring (DAM) system, a system originally developed for *Drosophila* studies and adapted for monitoring bee locomotor activity. Newly emerged, pathogen-free bees were selected for this study, as they lack circadian rhythms in early adulthood under constant conditions and develop them later [31]. To ensure data reliability, environmental conditions were kept stable throughout the experiment.

While automated systems efficiently collect data from isolated individuals, they do not account for sociobiological influences, which play a crucial role in shaping locomotor and circadian behaviors in bee colonies. For instance, young bees cohabiting with older foragers develop circadian rhythms at an earlier stage [27] and hive temperature, which is socially regulated, impacts circadian cycles [82]. Additionally, foraging bees lower their body temperature at night as part of endothermic regulation [83]. Although social factors were excluded, laboratory studies on isolated bees provide valuable insights into individual locomotor activity and circadian regulation [23,84,85]. This suggests that, under stable environmental conditions, genetic differences among subspecies were likely the primary factor influencing individual activity levels. The individual-based activity monitoring approach used in this study has important implications for interpreting behavioral responses to *V. ceranae* infection. In natural colony settings, behavioral patterns are significantly modulated by social factors including age polyethism, pheromonal communication, and collective immunity behaviors [86]. For example, infected foragers might modify their behavior due to social feedback from nestmates rather than direct physiological effects of infection. The contrasting activity patterns observed across subspecies at the individual level may manifest differently in colony contexts, where social regulatory mechanisms can either amplify or buffer these differences.

Future studies should extend these findings by examining how *V. ceranae* infection affects behavior within intact colonies, particularly focusing on the age-dependent division of labor and collective defensive responses. Automated tracking systems that monitor multiple individuals within observation hives would bridge the gap between our individual-level findings and colony-level resilience. Additionally, examining how the individual immune and behavioral responses identified here translate to colony-level outcomes, such as overwinter survival and population growth, would enhance the ecological relevance of these findings.

During the six-day *V. ceranae* infection period, the Syrian subspecies exhibited the highest activity levels. This occurred despite their elevated *V. ceranae* expression levels and the absence of a significant increase in antimicrobial peptide expression by day six, compared to other subspecies. Elevated vitellogenin transcript levels detected in the Syrian subspecies on the sixth day may indicate an adaptive response to *V. ceranae* infection, potentially supporting resilience during early-infection stages. However, the relationship between Vg expression and locomotor activity remains unclear and warrants further investigation.

The infected bees of the Muğla ecotype exhibited high *V. ceranae* mRNA levels. However, unlike the Syrian subspecies, their activity levels declined following infection. They also lacked a strong immune response. The absence of significant defensin and apidaecin upregulation in Muğla bees suggests a weaker humoral response to *V. ceranae* infection. This may contribute to higher susceptibility, but other factors, such as genetic background and metabolic constraints, may also play a role. The Carniolan subspecies exhibited a rapid humoral immune response during the first six days of infection, coinciding with elevated vitellogenin levels on day six. While infected Carniolan bees initially showed higher activity levels than the controls, this difference diminished by day 5 and reversed on day 6, suggesting a shift in physiological priorities as the infection progressed.

Caucasian bees exhibited the strongest immune response, which was accompanied by a reduction in activity levels after infection. The Yığılca ecotype exhibited a sharp rise in AMP gene expression. However, the relationship between immunity and activity remained unclear due to the variability in their activity patterns. The variability in locomotor activity among subspecies suggests that immune activation alone may not fully explain movement patterns following *V. ceranae* infection. Other factors, such as metabolic costs, oxidative stress, or neurophysiological alterations, may contribute to activity modulation, but further research is needed to confirm these potential mechanisms.

The concurrent decline in activity levels and increase in humoral immunity can be interpreted within the framework of cost–benefit analysis. *Vairimorpha* infections can alter host physiology and behavior, potentially linking the decline in activity to the energy-intensive production of antimicrobial peptides. Elevated immune responses can negatively affect colony performance by reducing lifespan and colony efficiency [87] and impairing cognitive capacity [88]. The decline in locomotor activity following the initial immune activation suggests that *V. ceranae* infection imposes a significant metabolic burden to honey bees. The energetic costs associated with antimicrobial peptide production and vitellogenin upregulation may lead to physiological trade-offs, ultimately impacting colony productivity and longevity. Our results suggest that certain subspecies may exhibit greater resilience against an energetically demanding disease, like *Vairimorpha*, possibly due to evolved pathogen resistance mechanisms adapted to their native environments. Future studies should further explore the metabolic impact of *Vairimorpha* infection, particularly in field conditions where additional stressors, such as nutritional deficits and pesticide exposure, may exacerbate energy demands. Genotypic effects on *Vairimorpha* susceptibility were observed in Denmark’s *Vairimorpha*-tolerance selection program [89]. However, a Europe-wide study found no such effect when evaluating different genotypes both within and outside their native regions [90]. Ref. [36] examined the rhythmic locomotor activity of three honey bee varieties (*gAHB*, Carnica, and Caucasica) using the DAM system, reporting endogenous rhythms close to 24 h at 35 °C and significant interindividual variability at 25 °C.

### 4.3. Practical Implications for Beekeeping and Breeding Programs

The subspecies-specific differences in immune and behavioral responses to *V. ceranae* infection observed in this study have several important practical implications for beekeeping and breeding programs. Disease management strategies should be tailored to colony genetic backgrounds. Caucasian, Carniolan, and Yığılca subspecies with strong AMP responses may benefit from practices enhancing innate immunity, such as immunostimulant supplementation. In contrast, Syrian and Muğla bees exhibited alternative resilience mechanisms, including elevated vitellogenin, suggesting management should support these mechanisms through nutritional interventions promoting vitellogenin production [91]. Our results guide selective breeding for *Vairimorpha* resistance. The coordinated expression of multiple AMPs in Caucasian, Carniolan, and Yığılca bees identifies valuable genetic resources, while Syrian bees maintained activity despite infection suggests incorporating their genetics might improve colony functionality under disease pressure. Breeding programs could develop hybrids combining strong AMP responses with vitellogenin-mediated resilience. Our findings underscore the importance of preserving locally adapted bee populations. The subspecies-specific responses documented likely reflect adaptations to regional pathogen pressures. This is particularly relevant in Turkey, which harbors exceptional honey bee diversity with five subspecies and multiple ecotypes.

The gene expression patterns identified can serve as molecular biomarkers for selective breeding, complementing traditional phenotypic selection and accelerating genetic improvement for disease resistance utilizing marker-assisted selection techniques [92]. Locomotor activity findings suggest behavioral monitoring can provide early indicators of infection, informing the development of tools detecting behavioral changes indicative of *Vairimorpha* infection, enabling timely intervention. Implementation requires a collaboration between researchers, breeders, and beekeepers to translate these findings into field-relevant practices, maximizing their impact on sustainable beekeeping in the face of ongoing *Vairimorpha* challenges.

### 4.4. Limitations and Future Directions

The use of two colonies per subspecies as biological replicates warrants consideration when interpreting subspecies-level differences. While we detected statistically significant differences in immune gene expression and locomotor activity, inter-colony variation within subspecies should not be overlooked. Honey bee colonies exhibit considerable genetic diversity, with queens mating with 12–20 drones, creating workers with different paternal lineages within a single colony [93]. This heterogeneity may influence pathogen responses, and inter-colony variation can arise from genetic composition, queen age, pathogen exposure history, and microbiome composition. Previous studies demonstrated that within-colony genetic diversity enhances disease resistance [94,95]. Our observed subspecies differences represent average responses of two colonies per subspecies, capturing some variation but not the full spectrum of possible responses. Future research should incorporate more colonies per subspecies from different geographic regions to better characterize immune and behavioral responses to *V. ceranae* infection. Studies examining the heritability of immune responses within subspecies would clarify the genetic basis of observed differences, potentially identifying specific loci or markers for breeding programs.

Our use of whole bee homogenates for gene expression analyses represents a methodological limitation when interpreting immune responses to *V. ceranae* infection. This approach provides an overview of systemic gene expression but cannot distinguish tissue-specific responses critical for understanding host–pathogen interaction dynamics. *V. ceranae* targets the midgut epithelium [18] while antimicrobial peptide production occurs primarily in the fat body [49] and cellular immune responses involving *eater* occur in hemolymph hemocytes [96]. Our observed differential gene expression likely reflects a complex interplay of tissue-specific responses that whole-body analysis cannot fully resolve. Previous studies show that *V. ceranae* immune responses vary across tissues. Ref. [65] found midgut-specific responses involving epithelial renewal and oxidative stress pathways not apparent in whole-body analyses. Ref. [71] observed that fat body-specific AMP expression provided more sensitive immune activation indicators than whole-body measurements. Vitellogenin, elevated in Syrian and Carniolan bees in our study, is primarily produced in the fat body but also in nurse bees’ hypopharyngeal glands [97], potentially reflecting functions beyond immunity. Future research should include tissue-specific analyses comparing immune gene expression in the midgut, fat body, and hemolymph. Such targeted approaches would provide a more nuanced understanding of immune gene expression dynamics during infection and may reveal subspecies-specific variations not detected in our whole-body analysis. Additionally, histological examination with in situ hybridization would help correlate pathogen load with localized immune activation.

## 5. Conclusions

Selective breeding of genetically distinct populations can enhance disease resistance. Local honey bee populations have exhibited greater survival rates against pathogens compared to introduced subspecies, emphasizing the need for caution when relocating or introducing non-native subspecies in regions, such as Turkey. This study underscores the importance of genetic background in influencing the immune and behavioral responses of *A. mellifera* subspecies to *V. ceranae* infection. The variations observed in antimicrobial peptide (AMP) expression, vitellogenin regulation, and locomotor activity across subspecies emphasize the importance of genotype in determining host resilience. These findings reinforce the necessity of region-specific disease management strategies and selective breeding programs for enhancing *Vairimorpha* tolerance.

The immune defense mechanisms of honey bees, including antimicrobial peptides, may contribute to their resilience and have potential applications in developing antibiotics and antifungal agents. This research examined the immune responses and locomotor behavior across various *A. mellifera* subspecies under *V. ceranae* challenge in a controlled laboratory environment. The upregulated immune response in some subspecies indicates protective immunity, offering insights into host–*Vairimorpha* interactions for better disease management. The observed variations in immune responses and behavioral traits across subspecies highlight the complexity of *Vairimorpha* infections and suggest that genetic factors play a crucial role in host–pathogen dynamics. Additional studies are required to elucidate the functions of specific *Vairimorpha* isolates and host strains in virulence dynamics, as well as to assess the impact of environmental stressors on immune and behavioral responses in field conditions. Future research should also focus on the interplay between immune activation, energy allocation, and environmental stressors in *Vairimorpha* infection dynamics. Investigating how nutritional status, pesticide exposure, and seasonal variations influence immune and locomotor responses will enhance our understanding of the processes driving *Vairimorpha* resilience.

## Data Availability

The data presented in this study are available on request from the corresponding author.
